# CEBPB as a prognostic biomarker and its association with immune cells in clear cell renal cell carcinoma

**DOI:** 10.1016/j.bbrep.2025.102231

**Published:** 2025-09-01

**Authors:** Yaoqiang Ren, Min Wei, Quanfa Tian, Wenke Guo

**Affiliations:** a Department of Urology Surgery, Fenyang Hospital of Shanxi Province, Lüliang, Shanxi, China; b Department of Thyroid Surgery, Fenyang Hospital of Shanxi Province, Lüliang, Shanxi, China

**Keywords:** ccRCC, CEBPB, Prognostic biomarker, Immune infiltration

## Abstract

Clear cell renal cell carcinoma (ccRCC) is a highly aggressive malignancy with a poor prognosis. This study examines the expression, prognostic significance, and immune association of CCAAT/enhancer-binding protein beta (CEBPB) in ccRCC. RNA sequencing data from The Cancer Genome Atlas (TCGA) and Genotype-Tissue Expression (GTEx) Project were analyzed using the STAR workflow and R software. Immunohistochemistry (IHC) validated CEBPB expression in ccRCC tissues. Functional enrichment analyses (Gene Ontology and Kyoto Encyclopedia of Genes and Genomes) and a protein-protein interaction (PPI) network (STRING and Cytoscape) were used to explore CEBPB-related pathways. Single-sample gene set enrichment analysis (ssGSEA) revealed significant correlations between CEBPB expression and the infiltration of 24 immune cell types. CEBPB was linked to immune-related pathways, including humoral immune response, leukocyte migration, and cytokine signaling. PPI analysis identified strong interactions with STAT3/EP300, highlighting its role in immune regulation. Cox regression analysis showed that high CEBPB expression is associated with poorer overall survival, supporting its potential as a prognostic biomarker. In conclusion, CEBPB plays a key role in shaping the immune microenvironment of ccRCC and may serve as a novel prognostic marker and therapeutic target.

## Highlights

• CEBPB overexpressed in 9 cancers, including ccRCC.
• IHC confirms high CEBPB expression in ccRCC.
• CEBPB expression in ccRCC higher than controls.
• CEBPB correlates with immune cells in ccRCC.
• High CEBPB expression links to poor prognosis.

## 1 Introduction

Renal cell carcinoma (RCC) is a prevalent malignancy of the urinary system, with clear cell renal cell carcinoma (ccRCC) being the most common subtype, accounting for 70–80 % of RCC cases [1]. In 2020, there were an estimated 431,288 new cases of kidney tumors globally, with 90 % being RCC. In 2022, China reported approximately 77,000 new cases and 46,000 deaths due to kidney cancer [2]. Despite advances in surgery, targeted therapies, and immunotherapies, the prognosis for advanced ccRCC remains poor, with a five-year survival rate below 10 % for metastatic disease [3]. This highlights the urgent need for novel biomarkers and therapeutic targets.

CEBPB, encoding CCAAT/enhancer-binding protein beta, is a transcription factor involved in immune response, cell proliferation, and differentiation [4]. Aberrant CEBPB expression has been implicated in various cancers, including breast, liver, and glioblastoma, suggesting its role in tumorigenesis [5,6]. For instance, CEBPB regulates genes involved in cell cycle progression and apoptosis in breast cancer and inflammatory pathways in liver cancer [[7], [8], [9]].

However, the role of CEBPB in ccRCC is not well understood. Given its critical functions in other cancers, CEBPB may play a significant role in ccRCC pathogenesis and progression. This study investigates CEBPB expression in ccRCC and its clinical significance using data from TCGA and HPA. We conducted statistical analyses, pathway enrichment, PPI network construction, immune infiltration analysis, survival analysis, and diagnostic efficacy evaluation. Our findings suggest CEBPB as a potential prognostic biomarker and therapeutic target in ccRCC.

## 2 Materials and methods

### 2.1 RNA sequencing data acquisition and bioinformatics analysis

RNA sequencing data from the clear cell renal cell carcinoma (ccRCC) project were obtained from The Cancer Genome Atlas (TCGA) (https://www.cancer.gov/ccg/research/genome-sequencing/tcga). The data were processed using the STAR workflow and quantified in transcripts per million (TPM). Clinical data were extracted and processed in R (version 4.2.1), excluding redundant and clinically irrelevant entries. Statistical analyses were performed using appropriate methods from the 'stats' and ‘car’ packages in R, with data visualization performed using ‘ggplot2'.

### 2.2 Subcellular localization of CEBPB protein

Using the HPA database (https://www.proteinatlas.org/), enter “CEBPB” and click “Search”, select the first result, “CCAAT/enhancer-binding protein beta” On the resulting page, navigate to the Subcellular tab, which provides detailed information on the subcellular localization of CEBPB within the cell.

### 2.3 Differential expression of CEBPB in pan-cancer analysis

RNA-seq data for 33 cancer types were downloaded and processed from the TCGA database (https://portal.gdc.cancer.gov) using the STAR workflow. Data were extracted in transcripts per million (TPM) format. Based on the characteristics of the data, the Wilcoxon rank sum test was selected for statistical analysis using the R packages 'stats' and ‘car’ (statistical analysis was performed only if the data met the required assumptions). Data visualization was performed using R (version 4.2.1) and the ‘ggplot2’ package.

### 2.4 Differential expression of CEBPB in clear cell renal cell carcinoma (ccRCC) and normal tissues

#### 2.4.1 Unpaired samples

RNA-seq data for the TCGA-ccRCC (clear cell renal cell carcinoma) project were downloaded and processed from the TCGA database using the STAR workflow. Data were extracted in TPM format. Samples lacking clinical information and duplicate data were removed. The data were log_2_-transformed using the formula log_2_(value + 1), and statistical analysis was performed using the *t*-test.

#### 2.4.2 Paired samples

RNA-seq data for the TCGA-ccRCC (clear cell renal cell carcinoma) project were downloaded and processed from the TCGA database using the STAR workflow. Data were extracted in TPM format, and paired tumor and adjacent normal samples were identified based on their corresponding IDs. Samples lacking clinical information were removed. The data were log2-transformed using the formula log_2_(value + 1), and statistical analysis was performed using the paired *t*-test.

### 2.5 Immunohistochemical staining

We performed immunohistochemical (IHC) staining on 12 paired samples of clear cell renal cell carcinoma (ccRCC) and adjacent normal tissues. The samples were fixed in 4 % paraformaldehyde, followed by dehydration, paraffin embedding, and sectioning. After dewaxing, antigen retrieval was performed. The sections were incubated overnight at 4 °C with a 1:100 dilution of anti-CEBPB primary antibody (Sanying Biotechnology Co., Ltd., Wuhan, China). Following washing, a 1:200 dilution of fluorescently labeled secondary antibody (Pumai Biotechnology Co., Ltd., Shanghai, China) was applied and incubated at room temperature for 50 min. Color development was performed using an IHC reagent kit (Zhongshu Jinqiao Biotechnology Co., Ltd., Beijing, China). Positive expression was indicated by a brownish-yellow color.

### 2.6 Clinical significance grouping analysis of CEBPB

Expression levels of CEBPB were analyzed across clinical variables using Wilcoxon rank sum tests and visualized with violin plots, employing log2(value + 1) transformed data.

### 2.7 Single-gene differential analysis of CEBPB and PPI network construction

DESeq package was used to analyze differential expression between high and low CEBPB expression groups, with results visualized using volcano plots. Based on the criteria of ‘p < 0.05′ and '|log_2_FoldChange| > 1′, a total of 1071 differentially expressed genes were identified from the single-gene differential analysis of CEBPB. The gene IDs were then converted into Entrez IDs for GO and KEGG enrichment analysis. The PPI network of CEBPB was constructed using the STRING database and visualized in Cytoscape.

### 2.8 Single-gene correlation analysis of CEBPB

Pearson correlation analysis was performed between CEBPB and protein-coding genes in TCGA.

### 2.9 Correlation analysis between CEBPB and immune infiltration

Spearman correlation analysis was performed between CEBPB expression and the immune infiltration matrix, with visualization using ggplot2. The co-expression of CEBPB with immune-related genes was also visualized.

### 2.10 Survival analysis

Survival analysis was conducted using the survival package, assessing the correlation between CEBPB expression and survival rates in ccRCC patients with Cox regression. Results were visualized using survminer and ggplot2.

### 2.11 Diagnostic prediction of CEBPB expression in ccRCC

The circlize package was used for chromosomal localization of CEBPB. ROC analysis was performed using the pROC and timeROC packages, with results visualized using ggplot2.

### 2.12 Construction and evaluation of prognostic nomograms

Independent clinical-pathological prognostic factors identified from Cox regression analysis were used to construct nomograms predicting 1, 3, and 5-year overall survival (OS) probabilities. Calibration curves were generated to validate the accuracy of the nomograms, with concordance indices calculated using bootstrapping.

## 3 Results

### 3.1 Subcellular localization of CEBPB and its differential expression in pan-cancer and clear cell renal cell carcinoma

**Fig. 1:**
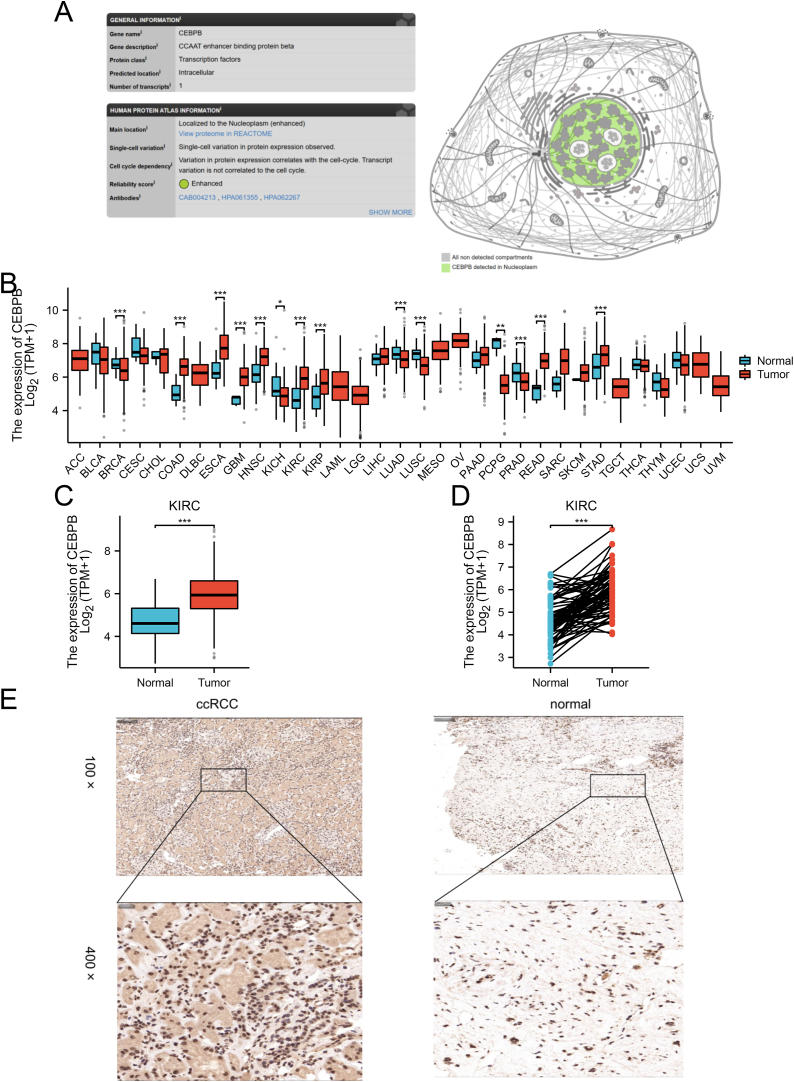
**Subcellular localization of CEBPB and its differential expression in clear cell renal cell carcinoma (ccRCC) and various other cancers.** (A) CEBPB protein is localized to the nucleoplasm. Data obtained from the Human Protein Atlas (HPA) database (https://www.proteinatlas.org/). (B) Differential expression of CEBPB across various cancers. (C) Difference in CEBPB expression between ccRCC patients and normal individuals. (D) Difference in CEBPB expression between ccRCC patients and paired adjacent normal samples. (E)IHC staining of CEBPB in cancer and paracancerous tissues. Representative images are shown. Score bars, 100, 20 μm ∗p < 0.05, ∗∗p < 0.01, ∗∗∗p < 0.001.

The CEBPB protein is localized to the nucleoplasm and exhibits single-cell expression variation that correlates with the cell cycle (Fig. 1A). Analysis of CEBPB expression in 33 cancer types using data from TCGA and GTEx revealed significant overexpression in nine cancer types: breast invasive carcinoma (BRCA) (p < 0.001), colon adenocarcinoma (COAD) (p < 0.001), esophageal carcinoma (ESCA) (p < 0.001), glioblastoma multiforme (GBM) (p < 0.001), head and neck squamous cell carcinoma (HNSC) (p < 0.001), kidney renal clear cell carcinoma (ccRCC) (p < 0.001), kidney renal papillary cell carcinoma (KIRP) (p < 0.001), rectum adenocarcinoma (READ) (p < 0.001), and stomach adenocarcinoma (STAD) (p < 0.001) (Fig. 1B). To further explore the relationship between CEBPB expression and ccRCC, gene expression data from 532 ccRCC patients and 72 normal controls with clinical information were collected from the TCGA database. The results indicated that CEBPB gene expression was significantly higher in ccRCC patients compared to normal controls, with statistical significance (p < 0.001, Fig. 1C). Subsequently, a paired comparison analysis was conducted using 72 ccRCC patients and 72 normal controls, revealing a significant increase in CEBPB expression in ccRCC patients (p < 0.001, Fig. 1D). Immunohistochemical (IHC) staining of clinical specimens was performed to validate the expression levels of CEBPB in clear cell renal cell carcinoma (ccRCC). The results indicated that CEBPB was expressed at higher levels in cancer tissues compared to adjacent non-cancerous tissues (Fig. 1E).

### 3.2 Correlation between CEBPB expression and clinicopathological features in clear cell renal cell carcinoma

**Fig. 2:**
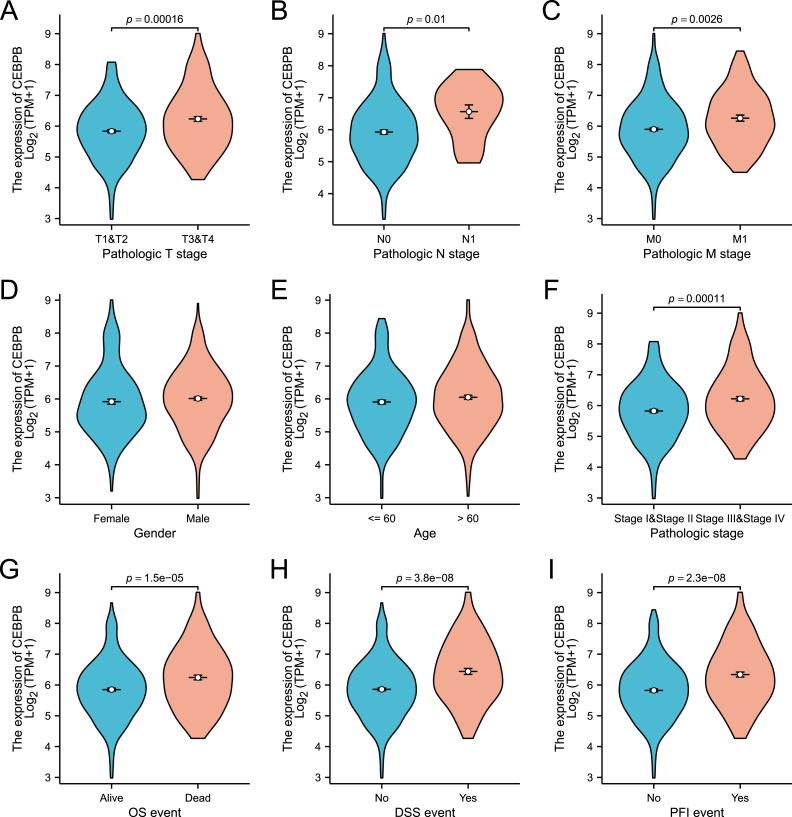
**Correlation between CEBPB expression levels and clinical-pathological characteristics of ccRCC patients.**(A–I) Visualization of the relationship between CEBPB expression and ten clinicopathological features including age, gender, T stage, N stage, M stage, pathological stage, overall survival (OS), disease-specific survival (DSS), and progression-free interval (PFI). Higher CEBPB expression is significantly associated with advanced T stage, N stage, M stage, pathological stage, and worse survival outcomes.

Baseline data for 532 ccRCC patients obtained from the TCGA database were statistically analyzed (Table 1). The data were divided into two groups based on the median expression level of CEBPB: 266 samples with low CEBPB expression and 266 samples with relatively high CEBPB expression. No significant differences were observed between the two groups in terms of gender (p = 0.057), age (p = 0.488), and laterality (p = 0.075). However, significant differences were noted in T stage (p = 0.015), N stage (p = 0.018), M stage (p = 0.019), and overall survival (OS) (p = 0.007). Subsequently, single-gene logistic regression analysis was performed, yielding results similar to those shown in Table 1, with no significant differences between the groups in terms of gender, age, and laterality. Further analysis revealed significant differences between T1/T2 and T3/T4 stages (p = 0.015, OR = 1.558 [1.090–2.226]), between N1 and N0 stages (p = 0.035, OR = 3.486 [1.093–11.118]), and between M0 and M1 stages (p = 0.019, OR = 1.795 [1.099–2.931]) (Table 2). Additionally, we visualized the relationship between CEBPB expression and ten clinicopathological features in ccRCC patients, including age, gender, T stage, N stage, M stage, pathological stage, Overall survival (OS), disease-specific survival (DSS), and progression-free interval (PFI) (Fig. 2). The results showed that high expression of CEBPB was significantly associated with T stage (p < 0.001) (Fig. 2A), N stage (p < 0.05) (Fig. 2B), M stage (p < 0.001) (Fig. 2C), pathological stage (p < 0.001) (Fig. 2F), OS events (p < 0.001) (Fig. 2G), DSS events (p < 0.001) (Fig. 2H), and PFI events (p < 0.001) (Fig. 2I). These data suggest that CEBPB is significantly upregulated in ccRCC and is associated with various clinical features.

**Table 1:** Correlation between CEBPB expression and clinicopathologic characteristics of ccRCC.

Characteristics	Low expression of CEBPB	High expression of CEBPB	P value
n	266	266	
T stage, n (%)			0.015
T1&T2	184 (34.6 %)	157 (29.5 %)	
T3&T4	82 (15.4 %)	109 (20.5 %)	
N stage, n (%)			0.026
N0	129 (50.4 %)	111 (43.4 %)	
N1	4 (1.6 %)	12 (4.7 %)	
M stage, n (%)			0.018
M0	226 (45.2 %)	195 (39 %)	
M1	31 (6.2 %)	48 (9.6 %)	
Pathologic stage			0.019
Stage I&Stage II	175 (33.1 %)	148 (28 %)	
Stage III&Stage IV	90 (17 %)	116 (21.9 %)	
Gender, n (%)			0.057
Female	104 (19.5 %)	83 (15.6 %)	
Male	162 (30.5 %)	183 (34.4 %)	
Age, n (%)			0.488
≤ 60	136 (25.6 %)	128 (24.1 %)	
>60	130 (24.4 %)	138 (25.9 %)	
OS event, n (%)			0.007
Alive	193 (36.3 %)	164 (30.8 %)	
Dead	73 (13.7 %)	102 (19.2 %)	
Laterality, n (%)			0.075
Left	115 (21.7 %)	135 (25.4 %)	
Right	151 (28.4 %)	130 (24.5 %)

**Table 2:** CEBPB expression associated with clinicopathologic characteristics (logistic regression) in ccRCC.

Characteristics	Total (N)	OR (95 % CI)	P value
Pathologic T stage (T3&T4 vs. T1&T2)	532	1.558 (1.090–2.226)	**0.015**
Pathologic N stage (N1 vs. N0)	256	3.486 (1.093–11.118)	**0.035**
Pathologic M stage (M1 vs. M0)	500	1.795 (1.099–2.931)	**0.019**
Age (>60 vs.≤60)	532	1.128 (0.803–1.585)	0.488
Gender (Male vs. Female)	532	1.415 (0.990–2.024)	0.057
Laterality (Right vs. Left)	531	0.733 (0.521–1.032)	0.075

### 3.3 CEBPB differential expression analysis, correlation analysis, and PPI network construction

**Fig. 3:**
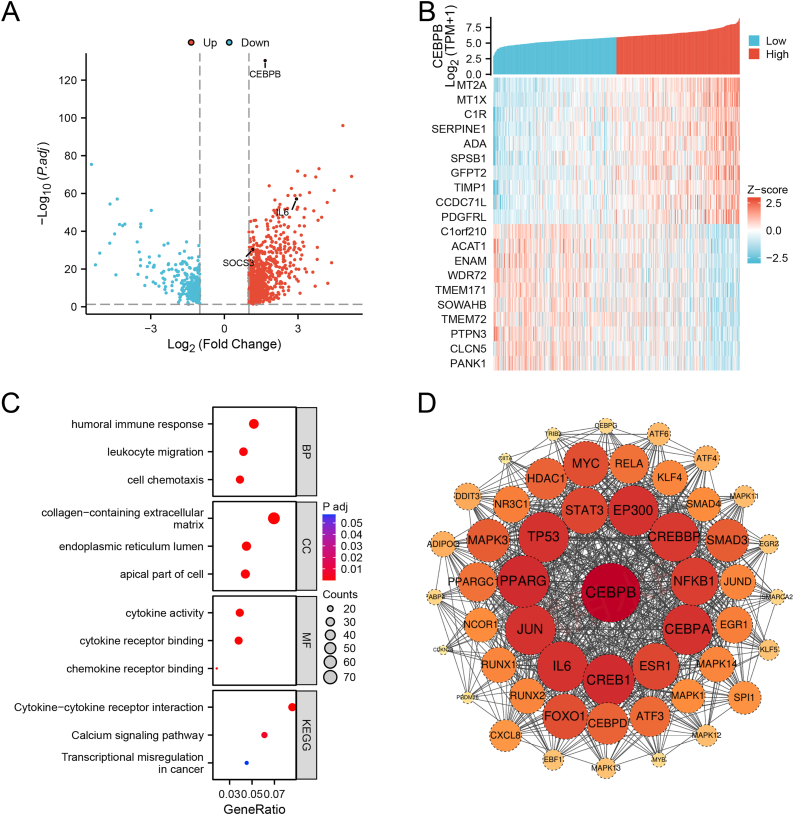
**CEBPB differential gene and related gene expression analysis, GOKEGG enrichment, and PPI network construction.**(A) Volcano plot of differentially expressed genes between high and low CEBPB expression groups from TCGA data, identifying 1071 DEGs.(B) Heatmap of the top 10 positively and negatively correlated genes with CEBPB expression.(C) After screening and ID conversion of differential genes associated with CEBPB expression, perform GOKEGG enrichment analysis.(D) Protein-protein interaction network of CEBPB, highlighting strong interactions with key proteins such as STAT3, EP300, and TP53.

The data were divided into high and low expression groups based on the median expression of CEBPB, with 266 samples in each group. Differential analysis of CEBPB was performed using DESeq2. The selection criteria included “Protein_coding,” "|logFC| > 1,” and “p.adj <0.05,” resulting in 1071 differentially expressed genes, comprising 792 upregulated genes and 279 downregulated genes (Fig. 3A). Subsequently, single-gene correlation analysis of CEBPB within the TCGA database was conducted. The data were filtered using the criteria "|Spearman correlation| > 0.3,” “p < 0.05,” and “protein_coding,” yielding 1314 correlated genes, including 1013 positively correlated genes and 301 negatively correlated genes. Spearman correlation coefficients were used for ranking, and the top 10 positive and negative correlated genes were selected for co-expression heatmap visualization. The results revealed that CEBPB expression was positively correlated with MT2A, MT1X, C1R, SERPINE1, PDGFRL, ADA, SPSB1, GFPT2, TIMP1, and CCDC71L, and negatively correlated with C1orf210, ACAT1, ENAM, WDR72, TMEM171, SOWAHB, TMEM72, PTPN3, CLCN5, and PANK1 (Fig. 3B). Further, the 1071 filtered differentially expressed genes were subjected to GO and KEGG functional enrichment analysis. Biological processes (BP) were enriched in the humoral immune response, leukocyte migration, and cell chemotaxis; cellular components (CC) were enriched in collagen-containing extracellular matrix, endoplasmic reticulum lumen, and apical part of cell; molecular functions (MF) were enriched in cytokine activity, cytokine receptor binding, and chemokine receptor binding; and KEGG pathways were enriched in Cytokine−cytokine receptor interaction, Calcium signaling pathway, and Transcriptional misregulation in cancer (Fig. 3C). The protein-protein interaction (PPI) network analysis revealed strong interactions between CEBPB and several proteins, including STAT3, EP300, CREBBP, NFKB1, CEBPA, ESR1, CREB1, IL6, JUN, PRARG, and TP53 (Fig. 3D).

### 3.4 Correlation analysis between CEBPB expression and immune cell abundance

**Fig. 4:**
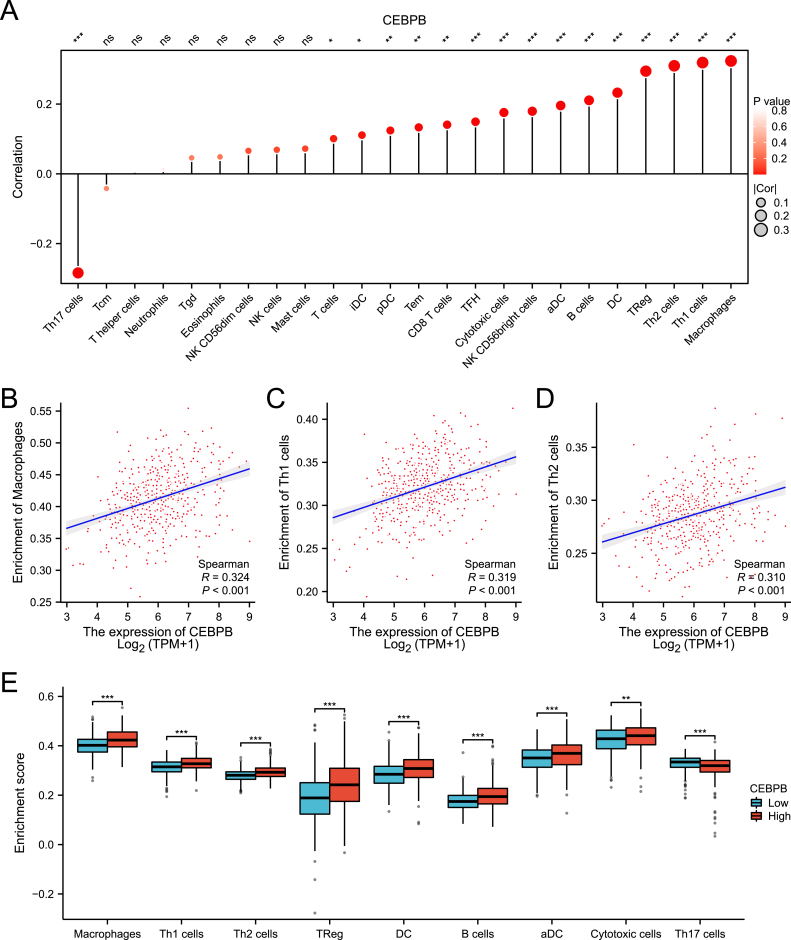
**Relationship between CEBPB expression and immune infiltration in the clear cell renal cell carcinoma (ccRCC) microenvironment.** (A) Correlation between CEBPB expression and the relative abundance of 24 immune cells. The size of the dots indicates the absolute value of Spearman's correlation coefficient R. (B–D) Correlation between CEBPB expression and infiltration levels of macrophages, Th1 cells, and Th2 cells. (E) Correlation between high and low CEBPB expression and the infiltration levels of aDC, cytotoxic cells, DC, macrophages, Th1 cells, Th2 cells, TReg, and Th17 cells. ∗p < 0.05, ∗∗p < 0.01, ∗∗∗p < 0.001.

Using the single-sample gene set enrichment analysis (ssGSEA) algorithm, we assessed the correlation between CEBPB expression levels and the abundance of 24 immune cell types, and visualized the results. As shown in Fig. 4A, the correlation between different immune cell abundances and CEBPB expression levels is represented by the size of the circles, with the color of the circle indicating the P-value. The distance from the baseline also reflects the strength of the correlation. Further analysis revealed a positive correlation between CEBPB expression and the abundance of macrophages (R = 0.324, p < 0.001), T helper 1 (Th1) cells (R = 0.319, p < 0.001), and T helper 2 (Th2) cells (R = 0.310, p < 0.001) (Fig. 4B–D). Additionally, we compared the immune cell abundances between the high and low CEBPB expression groups, and the results showed that in the high CEBPB expression group, the abundances of aDC, cytotoxic cells, DC, macrophages, Th1 cells, Th17 cells, Th2 cells, and TReg cells were significantly increased (p < 0.001) (Fig. 4E).

### 3.5 Impact of high CEBPB expression on prognosis of renal clear cell carcinoma (ccRCC) patients across different clinicopathological states

**Fig. 5:**
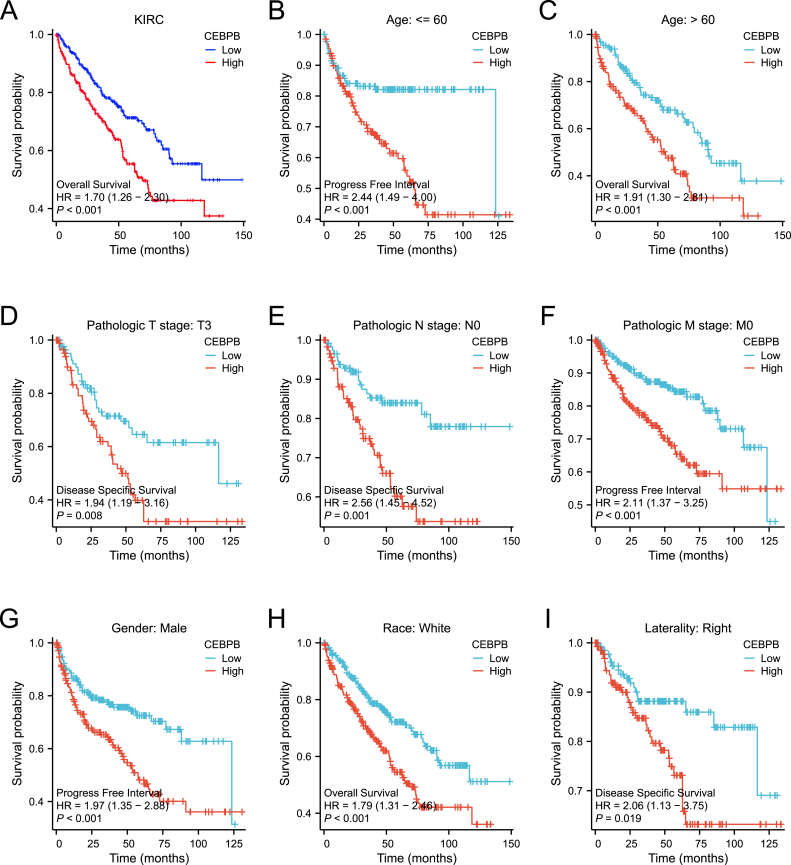
**Prognostic impact of CEBPB expression in ccRCC.**(A) Kaplan-Meier survival curve showing that high CEBPB expression is associated with poorer overall survival.(B–I) Subgroup analysis demonstrating that high CEBPB expression predicts worse prognosis across different clinical-pathological statuses.

Kaplan-Meier survival analysis based on data from the TCGA database revealed that high CEBPB expression is associated with poorer prognosis in ccRCC patients compared to low CEBPB expression (HR = 1.70, p < 0.001) (Fig. 5A) Subgroup analyses across various clinicopathological states further demonstrated that high CEBPB expression is significantly associated with poorer outcomes in the following patient groups:Patients younger than 60 years with a progression-free interval (PFI) (HR = 2.44, p < 0.001); Patients older than 60 years with overall survival (OS) (HR = 1.91, p < 0.001); Patients with pathological T3 stage with disease-specific survival (DSS) (HR = 1.94, p = 0.008); Patients with pathological N0 stage with disease-specific survival (DSS) (HR = 2.56, p = 0.001); Patients with pathological M0 stage with progression-free interval (PFI) (HR = 2.11, p < 0.001); Male patients with a progression-free interval (PFI) (HR = 1.97, p < 0.001); White patients with overall survival (OS) (HR = 1.79, p < 0.001); Patients with right-sided ccRCC with overall survival (OS) (HR = 2.06, p = 0.019). These findings are illustrated in Fig. 5B–E.

### 3.6 Diagnostic potential of CEBPB in ccRCC

**Fig. 6:**
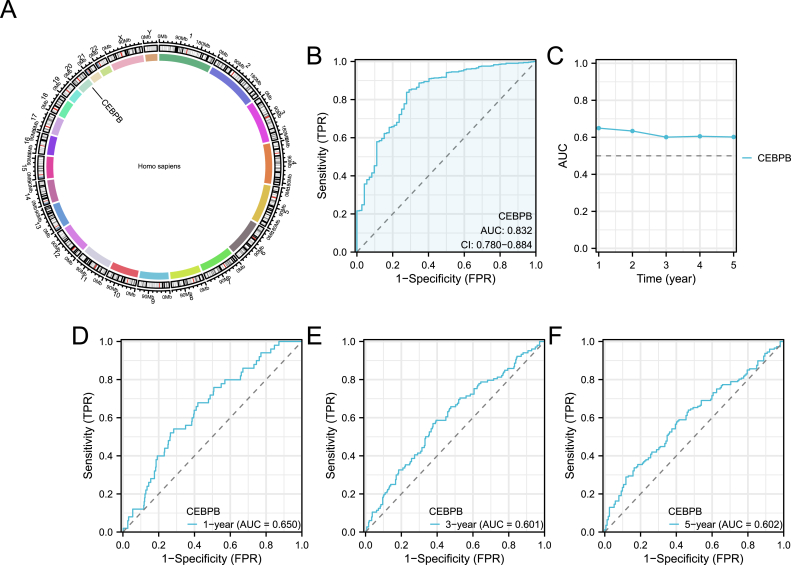
**Diagnostic efficacy of CEBPB in ccRCC.**(A) Chromosomal location of CEBPB on chromosome 20.(B) ROC curve showing the diagnostic performance of CEBPB for ccRCC with an AUC of 0.832.(C–F) Time-dependent ROC curves illustrating the predictive power of CEBPB for 1-, 3-, and 5-year overall survival.

CEBPB is located on chromosome 20 (Fig. 6A), It exhibits good diagnostic performance for clear cell renal cell carcinoma, with an AUC of 0.832 and a CI of 0.780–0.884 (Fig. 6B). CEBPB also demonstrates good diagnostic performance on Time-dependent ROC curves, predicting 1, 3, and 5-year overall survival rates, with AUC values ranging from 0.601 to 0.650 (Fig. 6C–F).

### 3.7 High expression of CEBPB is an independent risk factor for overall survival

To identify independent prognostic factors for ccRCC, including age, gender, T Stage, N Stage, M Stage, and CEBPB expression, univariate and multivariate Cox proportional hazards regression analyses were performed. Both univariate and multivariate analyses indicated that high CEBPB expression is an independent prognostic factor for overall survival (OS) in ccRCC patients (HR = 1.701, 95 % CI: 1.258–2.299, p < 0.001). Additionally, age (HR = 1.779, 95 % CI: 1.310–2.416, p < 0.001), N stage (HR = 3.395, 95 % CI [1.803–6.395], p < 0.001), M stage (HR = 4.343, 95 % CI [3.184–5.924], p < 0.001), and T stage (HR = 3.142, 95 % CI [2.323–4.251], p < 0.001) were also independent prognostic factors. These results are summarized in Table 3.

**Table 3:** Univariate and multivariate cox regression analysis of prognostic factors.

Characteristics	Total(N)	Univariate analysis		Multivariate analysis	
		Hazard ratio (95 % CI)	P value	Hazard ratio (95 % CI)	P value
Pathologic T stage	532				
T1&T2	341	Reference		Reference	
T3&T4	191	3.142 (2.323–4.251)	**< 0.001**	2.146 (1.368–3.369)	**< 0.001**
Pathologic N stage	256				
N0	240	Reference		Reference	
N1	16	3.395 (1.803–6.395)	**< 0.001**	1.856 (0.947–3.638)	0.072
Pathologic M stage	500				
M0	421	Reference		Reference	
M1	79	4.343 (3.184–5.924)	**< 0.001**	2.863 (1.773–4.622)	**< 0.001**
Age	532				
≤ 60	264	Reference		Reference	
>60	268	1.779 (1.310–2.416)	**< 0.001**	1.773 (1.156–2.718)	**0.009**
CEBPB	532				
Low	266	Reference		Reference	
High	266	1.701 (1.258–2.299)	**< 0.001**	1.351 (0.873–2.092)	0.177

### 3.8 Construction of prognostic nomogram and calibration curves

**Fig. 7:**
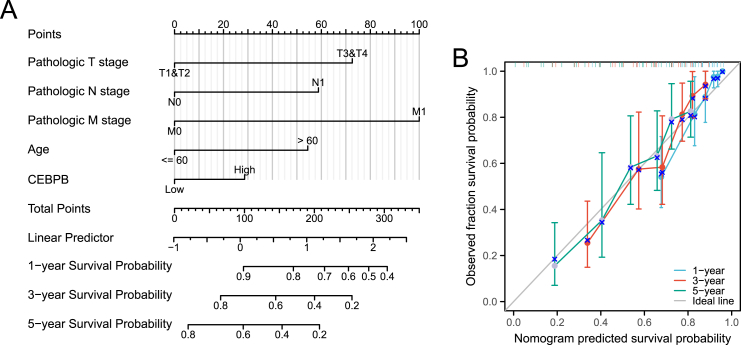
**Nomogram and calibration curves for predicting overall survival in ccRCC patients based on CEBPB expression and clinical factors.** (A) Nomogram integrating age, T stage, N stage, M stage, and CEBPB expression to predict 1-year, 3-year, and 5-year survival probabilities. (B) Calibration curves demonstrating the accuracy of the nomogram's predictions.

Independent prognostic factors, including age, T stage, N stage, M stage, and CEBPB expression, were used to construct a prognostic nomogram. Calibration curves were plotted to assess the nomogram's predictive accuracy. The relationships between the five clinicopathological variables and the 1-year, 3-year, and 5-year overall survival (OS) probabilities are visually presented (Fig. 7A). The 1-year, 3-year, and 5-year calibration curves show the agreement between our results and the predicted values, indicating satisfactory performance of this CEBPB-based nomogram. The concordance index (C-index) was 0.769, demonstrating excellent predictive accuracy (Fig. 7B).

## 4 Discussion

Renal clear cell carcinoma (ccRCC) is a predominant and highly aggressive subtype of kidney cancer, responsible for approximately 70–80 % of all renal malignancies [10]. This malignancy is characterized by high rates of metastasis and recurrence, which contribute to poor patient prognosis and limited treatment options [11]. Therefore, identifying molecular markers and therapeutic targets is essential for improving diagnosis, treatment, and overall survival in ccRCC patients.

In our study, we focused on the CEBPB gene, a transcription factor involved in inflammation, immune response, and cell differentiation [12]. Analysis of RNA sequencing data from the TCGA database revealed that CEBPB is significantly overexpressed in ccRCC tissues compared to normal tissues. To further validate this differential expression at the protein level, we employed immunohistochemistry (IHC) to confirm the expression trend of CEBPB between ccRCC and normal tissues, rather than performing a detailed quantitative analysis. As a semi-quantitative technique, IHC results may be influenced by staining conditions, tissue slide quality, and observer subjectivity [13]. Therefore, we relied primarily on mRNA expression analysis as a quantitative basis to ensure data stability and comparability. In future studies, we plan to expand the sample size and conduct a more detailed quantitative assessment of IHC staining intensity and color variations to provide more comprehensive supporting evidence.

Building on these findings, we explored the expression levels of CEBPB in clear cell renal cell carcinoma (ccRCC) and its clinical significance. During the data analysis, we observed differences in the role of CEBPB expression with respect to patient age across various analyses. In Fig. 2, statistical analysis indicated that there was no significant difference in the gene expression levels of CEBPB between patients aged ≤60 and > 60 years (p = 0.488). However, in the survival analysis shown in Fig. 5, high expression of CEBPB was associated with poorer survival outcomes across different age groups. This phenomenon can be attributed to the differences in statistical methods and research objectives. Fig. 2 used the Wilcoxon rank-sum test to evaluate the distribution of CEBPB expression across different clinical variables, showing that age itself does not significantly affect the expression level of CEBPB. In the Kaplan-Meier survival analysis in Fig. 5, the prognostic impact of high CEBPB expression was statistically significant across different age groups. Specifically, for patients ≤60 years, high CEBPB expression significantly decreased progression-free interval (PFI) (HR = 2.44, p < 0.001), while for patients >60 years, high CEBPB expression significantly decreased overall survival (OS) (HR = 1.91, p < 0.001). Therefore, our findings suggest that while CEBPB expression levels are not influenced by age, its impact on survival prognosis varies between different age groups. This may be related to age-associated changes in the immune microenvironment, tumor biological characteristics, or other potential mechanisms. Future studies could further integrate multi-omics data to explore the specific molecular mechanisms of CEBPB in patients of different ages, to better understand its true role in ccRCC progression.

Based on the results of GO and KEGG enrichment analysis of CEBPB-related differentially expressed genes (DEGs), we investigated its impact on the ccRCC immune microenvironment. This study employed the ssGSEA algorithm based on transcriptomic data to infer immune cell abundance [14], which effectively evaluates differences in immune cell proportions between samples. However, this method cannot directly reflect actual immune cell infiltration. Therefore, our findings are more likely to indicate that CEBPB participates in the regulation of the ccRCC immune microenvironment rather than directly driving immune infiltration. Since this study relies on publicly available database data, experimental validation is still required to elucidate the specific mechanisms underlying CEBPB's role in immune infiltration. Moreover, CEBPB may influence the immune microenvironment through the regulation of multiple immune-related signaling pathways, but this study did not delve into its precise regulatory mechanisms [15]. Future research could integrate in vitro immune cell co-culture experiments or animal models to further validate its function and clarify its impact on immune cell interactions within the tumor microenvironment.

Our protein-protein interaction (PPI) network analysis identified significant interactions between CEBPB and STAT3, a key transcription factor involved in oncogenesis, immune evasion, and inflammation [16]. The co-expression and interaction of CEBPB and STAT3 may enhance oncogenic processes in ccRCC, contributing to its aggressive nature by shaping the tumor microenvironment through immune cell recruitment and activation [17,18]. Additionally, EP300, a histone acetyltransferase, was found to interact with CEBPB, suggesting a cooperative mechanism that amplifies CEBPB-driven transcriptional activation of tumor-promoting and immune-related genes [19]. This interaction could modulate the acetylation of histones and non-histone proteins, altering gene expression essential for tumor progression and survival [20]. Targeting the CEBPB-STAT3 and CEBPB-EP300 axes may provide novel therapeutic strategies for ccRCC.

Moreover, we developed a nomogram based on CEBPB expression levels and clinical pathological characteristics (age, T, N, M staging) to predict the 1-year, 3-year, and 5-year overall survival (OS) of patients. However, the analysis revealed that the contribution of CEBPB in this model was relatively low, with its score being significantly lower than that of TNM staging and patient age. The independent predictive ability of CEBPB in multivariate analysis was relatively weak. Although univariate analysis showed that high expression of CEBPB was associated with poor prognosis, its independent predictive capacity diminished when stronger clinical prognostic factors (such as TNM staging and age) were included. This may suggest that the influence of CEBPB is relatively indirect, potentially affecting tumor progression through other mechanisms rather than directly determining the patient's survival time. CEBPB may be more involved in the biological processes of the tumor rather than being a primary determinant of survival. Previous studies have shown that CEBPB plays an important role in cell proliferation, inflammation regulation, and shaping the immune microenvironment, but it may mainly affect tumor invasiveness or treatment response (for example, by influencing tumor-associated macrophages or inflammatory pathways) rather than directly determining long-term survival. In contrast, TNM staging more intuitively reflects tumor burden and disease progression, which is why it carries greater weight in the nomogram. Biological differences between individual patients may influence the predictive value of CEBPB across the entire cohort. This study was based on the TCGA database, which includes patients with varying stages, molecular subtypes, and treatment regimens. The prognostic role of CEBPB may vary across different subgroups. Therefore, the overall contribution of CEBPB across the entire study cohort was relatively low. Future research will include stratified analyses of different subgroups (such as early vs. late-stage patients or different genetic mutation statuses) to further explore the clinical value of CEBPB in specific patient populations.

## 5 Conclusion

In conclusion, our bioinformatics analysis indicates that the expression levels of CEBPB are significantly elevated in clear cell renal cell carcinoma (ccRCC), which is consistent with existing studies. Our findings reveal a correlation between increased CEBPB expression and unfavorable clinical outcomes, such as reduced survival rates, suggesting that CEBPB could be a potential prognostic biomarker for ccRCC. Although these results are based primarily on computational analysis and lack experimental validation, they provide valuable insights into the role of CEBPB in the tumor immune microenvironment. Further experimental validation and clinical studies are necessary to confirm the clinical utility of CEBPB as a prognostic marker and therapeutic target.

Based on the current bioinformatics analysis, the prognostic nomogram we developed shows considerable potential for survival prediction in ccRCC patients and may provide important clinical guidance. Future research should integrate laboratory experiments and clinical data to further validate the biological function of CEBPB and its role in ccRCC. Nevertheless, the current findings lay a solid foundation for the clinical application of CEBPB and offer significant directions for future research [21].

## CRediT authorship contribution statement

**Yaoqiang Ren:** Writing – original draft. **Min Wei:** Writing – review & editing. **Quanfa Tian:** Formal analysis. **Wenke Guo:** Supervision.

## Funding

This work was supported by the Key Research and Development Program of Lvliang (No. 2022SHFZ12) and the Scientific Research Project of Shanxi Provincial Health Commission (No. 2024222).

## Declaration of competing interest

The authors declare that they have no conflicts of interest regarding the publication of this manuscript.

## Figures and Tables

**Table:** 

Abbreviations	Full name
ccRCC	Clear Cell Renal Cell CarcinomaBreast Differentially
DEGs	Expressed Genes
GEO	Gene Expression Omnibus
GO	Gene Ontology
KEGG	Kyoto Encyclopedia of Genes and Genomes
IL-6	Interleukin 6
RNA-seq	RNA sequencing
STAT3	Signal Transducer and Activator of Transcription 3
TCGA	The Cancer Genome Atlas
TME	Tumor Microenvironment
CEBPB	CCAAT/Enhancer Binding Protein Beta
FDR	False Discovery Rate
